# The generation of genuine quadripartite Einstein–Podolsky–Rosen steering in an optical superlattice

**DOI:** 10.1038/s41598-023-48626-z

**Published:** 2023-12-01

**Authors:** Y. R. Shen, T. H. Chen, S. L. Liang, X. Y. Cheng, J. W. Lv, Y. X. Jiang, L. Cheng, Y. B. Yu, G. R. Jin, A. X. Chen

**Affiliations:** Key Laboratory of Optical Field Manipulation of Zhejiang Province, Department of Physics, Zhejiang Sci-Tech University, Hangzhou, 310018 China

**Keywords:** Quantum optics, Quantum information

## Abstract

Einstein–Podolsky–Rosen (EPR) steering is a quantum effect based on quantum entanglement and it is the key resource for building quantum networks because of its useful properties. Based on the criterion for genuine multipartite EPR steering, the genuine quadripartite EPR steering is confirmed and it can be generated by a spontaneous parametric down-conversion cascaded process with two sum-frequency generations in an optical superlattice. This occurs either below the oscillation threshold and without oscillation threshold. The influence of the parameters of cascaded nonlinear process on the quadripartite EPR steering among signal, idler, and two sum-frequency beams are also discussed. Choosing appropriate nonlinear parameters can achieve good quadripartite quantum steering. This scheme of the generation of genuine quadripartite EPR steering has potential applications in quantum communication and computing.

## Introduction

In 1935, the concept of quantum steering was proposed by Schrödinger^1^ to formalise the paradox discussed by Einstein, Podolsky and Rosen^2^. Einstein–Podolsky–Rosen (EPR) steering emerges the fact that one of the parties can steer the state of the other distant party by utilizing their shared entanglement state. Nevertheless, Schrö dinger’s early works didn’t receive much attention. Recently, quantum steering attracted renewed interest because a formal definition was proposed and systematic criteria were developed by Wiseman *et al.*^3^.

He and Reid^4^ formalized the concept of genuine N-partite EPR steering and put forward the criterion for multipartite EPR steering. Teh *et al.*^5,6^ derived inequalities sufficient to detect the genuine N-partite steering of N distinct systems. Skrzypczyk *et al*.^7^ demonstrated every pure entangled state is maximally steerable and the antisymmetric subspace is maximally steerable for all dimensions. Kogias *et al*.^8^ introduced a computable measure of steering for arbitrary bipartite Gaussian states of continuous variable systems. By using optical networks and efficient detection, Armstrong *et al* .^9^ presented experimental observations of multiparty EPR steering and the genuine entanglement of three intense optical beams. Ji *et al*.^10^ showed that only a negative partial-transpose state can manifest quantum steerability through Gaussian measurements in relation to the Peres conjecture. Li *et al*.^11^ experimentally demonstrated genuine Quadrapartite EPR steering for the first time and applications to universal one-way quantum computing. Zeng * et al*.^12^ investigated the steering effect for the first time by encoding with orbital angular momentum of photon. Shi *et al*.^13^ derived a series of conditions to determine whether the EPR steering exists or not and further proved that the EPR steering can be tested by the specific initial state. Tripartite EPR steering in four-wave mixing of Rubidium atoms was confirmed by Liu *et al*.^14^. Multipartite quantum entanglement can be generated by coupled intracavity nondegenerate parametric down-conversion^15,16^ and cascaded nonlinear process^17,18^. Quantum steering is different from quantum entanglement. It can realize one-way quantum control and complete some tasks that quantum entanglement cannot complete. Multipartite quantum steering can also be generated by cascaded nonlinear processes. The genuine tripartite EPR steering among pump, second-harmonic, and third-harmonic was demonstrated by Liu *et al*.^19^. Genuine tripartite EPR steering in cascaded nonlinear process of quasi-phase-matching fourth-harmonic generation^20^ and the genuine tripartite EPR steering in spontaneous parametric down-conversion cascaded with a sum-frequency generation^21^ were also investigated. Liang *et al*.^22^ demonstrated the generation of genuine quadripartite quantum steering by an injected signal optical parametric oscillator cascaded with a sum-frequency process. However, the genuine quadripartite EPR steering in spontaneous parametric down-conversion cascaded with two sum-frequency generations has not been investigated.

In the paper, we propose a scheme to generate genuine quadripartite EPR steering by spontaneous parametric down-conversion (SPDC) cascaded with two sum-frequency processes in an optical cavity. The threshold characteristics of the cascaded nonlinear process are also discussed.

## Methods

**Figure 1:**
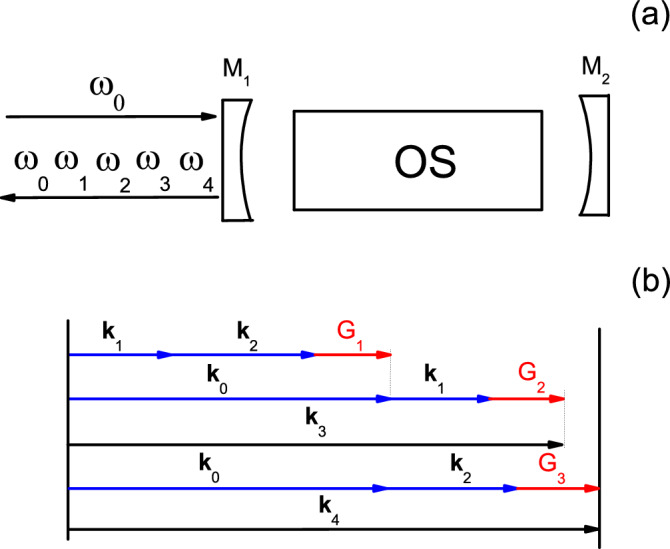
(**a**) Sketch of the one-sided optical oscillator cavity. (**b**) QPM schematic for the cascaded nonlinear interactions.

By using a one-sided optical oscillator cavity which can be seen in Fig. 1a, we investigate the cascaded nonlinear interactions which coupled with each other through quasi-phase-matching (QPM)^23^ scheme. The pump with the frequency $\omega _0$ enters the cavity from the left. The two beams of signal with the frequency of $\omega _1$ and idler with the frequency of $\omega _2$ are generated by the first nonlinear process of SPDC in the optical superlattice. The third beam with the frequency of $\omega _3$ is produced by a cascaded sum-frequency generation process between pump and signal. Finally, the forth beam with the frequency of $\omega _4$ is produced by the second cascaded sum-frequency generation process between pump and idler. We assumed that the pump (frequency at $\omega _{0}$), signal (frequency at $\omega _{1}$), idler (frequency at $\omega _{2}$), and two sum-frequency (frequencies at $\omega _{3}$ and $\omega _{4}$) modes are all perfectly resonant in the cavity and output the cavity through the coupling mirror M$_1$. These three cascaded nonlinear processes can be achieved through QPM technology. The schematic diagram of QPM is shown in Fig. 1b. ${\textbf {k}} _{0}$, ${\textbf {k}} _{1}$, ${\textbf {k}} _{2}$, ${\textbf {k}} _{3}$, and ${\textbf {k}} _{4}$ are the corresponding wave vectors of pump, signal, idler, and two sum-frequency fields, respectively. ${\textbf {G}}_{1}$, ${\textbf {G}}_{2}$, and ${\textbf {G}}_{3}$ are three reciprocals provided by the optical superlattice (OS). They satisfy the following relationships.

1 $$\begin{aligned} {\textbf {k}} _{0}= & {} {\textbf {k}} _{1}+{\textbf {k}} _{2}+{\textbf {G}} _{1},\nonumber \\ {\textbf {k}} _{3}= & {} {\textbf {k}} _{0}+{\textbf {k}} _{1}+{\textbf {G}} _{2},\nonumber \\ {\textbf {k}} _{4}= & {} {\textbf {k}} _{0}+{\textbf {k}} _{2}+{\textbf {G}} _{3}. \end{aligned}$$

The Hamiltonian of the cascaded nonlinear process interaction is

2 $$\begin{aligned} \mathscr {H}_{I}=i\hbar (\kappa _{1}\hat{a}_{0}\hat{a}_{1}^{+}\hat{a} _{2}^{+}+\kappa _{2}\hat{a}_{0}\hat{a}_{1}\hat{a}_{3}^{+}+\kappa _{3}\hat{a} _{0}\hat{a}_{2}\hat{a}_{4}^{+})+h.c., \end{aligned}$$

where $\hat{a}_{i}$ is the annihilation operator of the optical field with the frequency of $\omega _{i}$. $\kappa _{i}$ is the real nonlinear interaction coupling constant^24^. The first term is the SPDC process, while the second and third terms are the two cascaded sum-frequency processes of pump and signal and idle, respectively. The cavity pumping is given by $\mathscr {H}_{pump}=i\hbar \epsilon \hat{a}_{0}^{+}+h.c.,$ where $\epsilon$ is the real pump amplitude.

The master equation of this cascaded nonlinear processes is

3 $$\begin{aligned} \frac{d\hat{\rho }}{dt}=-\frac{i}{\hbar }[\mathscr {H}_{I}+\mathscr {H}_{pump}, \hat{\rho }]+\overset{4}{\underset{i=0}{\sum }}\mathscr {L}_{i}\hat{\rho }, \end{aligned}$$

where $\mathscr {L}_{i}\hat{\rho }=\gamma _{i}(2\hat{a}_{i}\hat{\rho }\hat{a}_{i}^{+}- \hat{a}_{i}^{+}\hat{a}_{i}\hat{\rho }-\hat{\rho }\hat{a}_{i}^{+}\hat{a}_{i}),$. $\gamma _{i}$ is the cavity loss rate.

By mapping the master equation onto Fokker–Planck equation (FPE) in the positive-*P* representation^25,26^, one can obtain five stochastic differential equations

4 $$\begin{aligned} \frac{d\alpha _{0}}{dt}= & {} \varepsilon -\gamma _{0}\alpha _{0}-\kappa _{1}\alpha _{1}\alpha _{2}-\kappa _{2}\alpha _{1}^{+}\alpha _{3}-\kappa _{3}\alpha _{2}^{+}\alpha _{4}+\sqrt{-2\kappa _{2}\alpha _{3}}(\eta _{1}+i\eta _{2})+\sqrt{-2\kappa _{3}\alpha _{4}}(\eta _{3}+i\eta _{4}), \nonumber \\ \frac{d\alpha _{1}}{dt}= & {} -\gamma _{1}\alpha _{1}+\kappa _{1}\alpha _{0}\alpha _{2}^{+}-\kappa _{2}\alpha _{0}^{+}\alpha _{3}+\sqrt{-2\kappa _{2}\alpha _{3}}(\eta _{1}-i\eta _{2})+\sqrt{2\kappa _{1}\alpha _{0}}(\eta _{5}+i\eta _{6}), \nonumber \\ \frac{d\alpha _{2}}{dt}= & {} -\gamma _{2}\alpha _{2}+\kappa _{1}\alpha _{0}\alpha _{1}^{+}-\kappa _{3}\alpha _{0}^{+}\alpha _{4}+\sqrt{-2\kappa _{3}\alpha _{4}}(\eta _{3}-i\eta _{4})+\sqrt{2\kappa _{1}\alpha _{0}}(\eta _{5}-i\eta _{6}), \nonumber \\ \frac{d\alpha _{3}}{dt}= & {} -\gamma _{3}\alpha _{3}+\kappa _{2}\alpha _{0}\alpha _{1}, \nonumber \\ \frac{d\alpha _{4}}{dt}= & {} -\gamma _{4}\alpha _{4}+\kappa _{3}\alpha _{0}\alpha _{2}, \end{aligned}$$

where $\eta _{i}(t)(i=1$,2,3,4,5,6) are the Gaussian noise terms with the properties $<\eta _{i}(t)>=0$ and $<\eta _{i}(t)\eta _{j}(t^{\prime })>$ $=\delta _{ij}\delta (t-t^{^{\prime }})$.

In the following, in order to solve the differential equation system above, we will use a linearization method. One can regard the positive-*P* variables as $\alpha _{i}=A_{i}+\delta \alpha _{i}$ $(i=0,1,2,3,4)$, where $A_{i}$ is steady-state expectation values, and $\delta \alpha _{i}$ is delta-correlated Gaussian fluctuation terms. After add the coupling equations of Eq. (4), the equations of motion for the system can be rewritten as,

5 $$\begin{aligned} d\delta \tilde{\alpha }=-\textbf{A}\delta \tilde{\alpha }dt+\textbf{B}dW, \end{aligned}$$

with $\delta \tilde{\alpha }=[\delta \alpha _{0},\delta \alpha _{0}^{+},\delta \alpha _{1},\delta \alpha _{1}^{+},\delta \alpha _{2},\delta \alpha _{2}^{+},\delta \alpha _{3},\delta \alpha _{3}^{+},\delta \alpha _{4},\delta \alpha _{4}^{+}]^{\textrm{T}}.$ $\textbf{A}$ is the drift matrix, $\textbf{B}$ contains the steady-state solutions of noise terms, and *dW* is a vector of Wiener increments^26^. One can obtain the intracavity spectra as

6 $$\begin{aligned} \textbf{S}(\omega )=(\textbf{A}+i\omega \textbf{I})^{\mathrm {-1}}\textbf{B}\textbf{B}^{ \textrm{T}}( \textbf{A}^{\textrm{T}}-i\omega \textbf{I})^{\mathrm {-1}}, \end{aligned}$$

where $\omega$ and $\textbf{I}$ correspond to the Fourier analysis frequency and the identity matrix, respectively. In this case, related to the standard input-output relationship^27^, the output spectra may be calculated.

It is very important to note that the situation we consider is the modes without including pump mode in the following analysis. The stationary solutions without the noise terms are divided into two different sets on the basis of whether a oscillation threshold exists or not.

The steady-state solutions can also be found from the stochastic differential equations. We find that the system has a threshold^28^

7 $$\begin{aligned} \varepsilon _{c}=\sqrt{\frac{\gamma _{3}\gamma _{4}\kappa _{1}^{2}-\gamma _{2}\gamma _{4}\kappa _{2}^{2}-\gamma _{1}\gamma _{3}\kappa _{3}^{2}-\sqrt{ (\gamma _{3}\gamma _{4}\kappa _{1}^{2}-\gamma _{2}\gamma _{4}\kappa _{2}^{2}-\gamma _{1}\gamma _{3}\kappa _{3}^{2})^{2}-4\gamma _{1}\gamma _{2}\gamma _{3}\gamma _{4}\kappa _{2}^{2}\kappa _{3}^{2}}}{2\kappa _{2}^{2}\kappa _{3}^{2}}}. \end{aligned}$$

for $$\kappa _{1}\geqslant \sqrt{\frac{\gamma _{2}}{\gamma _{3}}}\kappa _{2}+\sqrt{\frac{\gamma _{1}}{\gamma _{4}}}\kappa _{3}$$.

However, the system has no threshold for $\kappa _{1}<\sqrt{\frac{\gamma _{2}}{\gamma _{3}}}\kappa _{2}+ \sqrt{\frac{\gamma _{1}}{\gamma _{4}}}\kappa _{3}$ and the pump has a critical value^28^

8 $$\begin{aligned} \varepsilon _{c}^{^{\prime }}=\sqrt{\frac{\gamma _{3}\gamma _{4}\kappa _{1}^{2}-\gamma _{2}\gamma _{4}\kappa _{2}^{2}-\gamma _{1}\gamma _{3}\kappa _{3}^{2}+\sqrt{(\gamma _{3}\gamma _{4}\kappa _{1}^{2}-\gamma _{2}\gamma _{4}\kappa _{2}^{2}-\gamma _{1}\gamma _{3}\kappa _{3}^{2})^{2}-4\gamma _{1}\gamma _{2}\gamma _{3}\gamma _{4}\kappa _{2}^{2}\kappa _{3}^{2}}}{ 2\kappa _{2}^{2}\kappa _{3}^{2}}}. \end{aligned}$$.

When $\varepsilon \le \epsilon _{c}$ and $\varepsilon \le \varepsilon _{c}^{^{\prime }}$, the system is stable and the the linearization method is effective. In the next section, we will discuss the multipartite quantum steering within these ranges.

## Results

We defined $X_{i}=(\alpha _i+\alpha _i^{\dagger })/2$ and $Y_{i}=(\alpha _i-\alpha _i^{\dagger })/2i$ as the quadrature amplitude and phase component, respectively. Based on the criterion for genuine multipartite EPR steering^4–6^, a set of inequalities is given as

9 $$\begin{aligned} V_{1}= & {} \Delta (X_{1}-X_{2})\Delta (Y_{1}+Y_{2}+Y_{3}+Y_{4})<1 \nonumber \\ V_{2}= & {} \Delta (X_{2}-X_{3})\Delta (Y_{1}+Y_{2}+Y_{3}+Y_{4})<1 \nonumber \\ V_{3}= & {} \Delta (X_{3}-X_{4})\Delta (Y_{1}+Y_{2}+Y_{3}+Y_{4})<1 \nonumber \\ V_{4}= & {} \Delta (X_{4}-X_{1})\Delta (Y_{1}+Y_{2}+Y_{3}+Y_{4})<1. \end{aligned}$$

EPR steering of system *i* will be confirmed when the values of $V_{i}<1$. Such as $V_{1}<1$ shows the steering exists between the different bipartitions {1,234}, 134,2, {13,24}, or {14,23}. $V_{2}<1$ shows the steering exists between the different bipartitions^5,6^ {2,134}, {3, 124}, {12,34}, or {13,24}. $V_{3}<1$ shows the steering exists between the different bipartitions {3,124}, {4, 123}, {13,24}, or {14,23}. $V_{4}<1$ shows the steering exists between the different bipartitions {1,234}, {4,123}, {12,34}, or {13,24}. Remarkably, genuine quadripartite EPR steering will be verified as long as^4–6^

10 $$\begin{aligned} V_{t}=V_{1}+V_{2}+V_{3}+V_{4}<1. \end{aligned}$$

In the following, the quadripartite EPR steering will be discussed both below the threshold and without the threshold, respectively.

### Below the threshold

**Figure 2:**
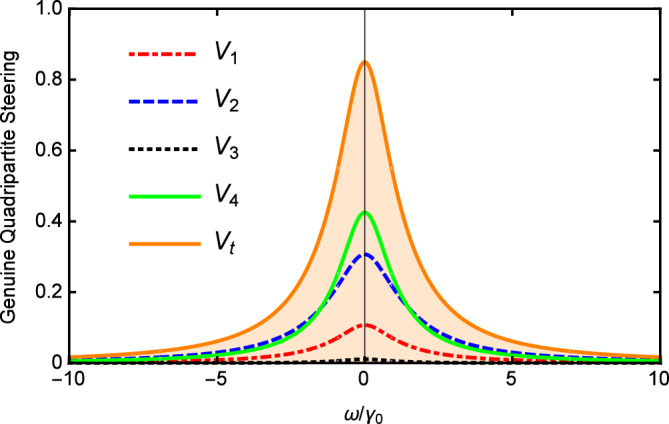
$V_{i}$ and $V_{t}$ versus the normalized analysis frequency $\Omega$ below the threshold with $\gamma _{0}=\gamma _{1}=0.02$, $\gamma _{2}=2 \gamma _{1}$, $\gamma _{3}=4\gamma _{1}$, $\gamma _{4}=5\gamma _{1}$, $\kappa _{1}=5\gamma _{1}$ , $\kappa _{2}=0.5\kappa _{1}$, and $\kappa _{3}=0.2\kappa _{1}$.

Figure 2 depicts $V_{i}$ and $V_{t}$ versus the normalized analysis frequency $\Omega$ with $\varepsilon =0.08\varepsilon _{c}$, $\gamma _{0}=\gamma _{1}=0.02$, $\gamma _{2}=2\gamma _{1}$, $\gamma _{3}=4\gamma _{1}$, $\gamma _{4}=5\gamma _{1}$, $\kappa _{1}=5\gamma _{1}$, $\kappa _{2}=0.5\kappa _{1}$, and $\ \kappa _{3}=0.2\kappa _{1}$. As shown in Fig. 2, the value of $V_{i}$ is below 1, and most importantly, $V_{t}$ is also below 1 in the whole range of $\Omega$. It shows that the genuine quadripartite EPR steering can be generated in our scheme based on cascaded nonlinear processes.

**Figure 3:**
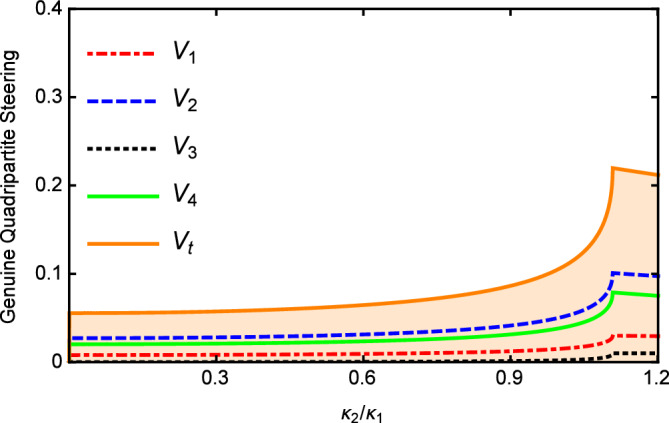
The values of $V_{i}$ and $V_{t}$ versus $\kappa _{2}/\kappa _{1}$, with $\gamma _{0}=\gamma _{1}=0.02$, $\gamma _{2}=2\gamma _{1}$, $\gamma _{3}=4\gamma _{1}$, $\gamma _{4}=5\gamma _{1}$, $\kappa _{1}=5\gamma _{1}$, and $\ \kappa _{3}=0.2 \kappa _{1}$ below the threshold.

In Fig. 3, we show the results of $V_{i}$ and $V_{t}$ versus the nonlinear coupling parameter $\kappa _{2}/\kappa _{1}$ with $\varepsilon =0.08\varepsilon _{c}$, $\gamma _{0}=\gamma _{1}=0.02$, $\gamma _{2}=2\gamma _{1}$, $\gamma _{3}=4\gamma _{1}$, $\gamma _{4}=5\gamma _{1}$, $\kappa _{1}=5\gamma _{1}$, $\kappa _{3}=0.2\kappa _{1}$, and $\omega =5\gamma _{0}$. It can be seen that the values of $V_{i}$ and $V_{t}$ increase slowly as the increase of the nonlinear coupling parameter. When $\kappa _{2}>\kappa _{1}$, $V_{i}$ and $V_{t}$ slowly decrease with the increase of $\kappa _2$. However, limited by the threshold condition, $\kappa _2$ cannot continue to increase. Below the threshold, the change of $\kappa _{2}/\kappa _{1}$ has little effect on multipartite quantum steering which is different from the case above the threshold. Nevertheless, $V_{i}$ and $V_{t}$ are all below 1 in the whole range of Fig. 3 which is sufficient to demonstrate that the genuine quadripartite EPR steering can be produced in our scheme.

**Figure 4:**
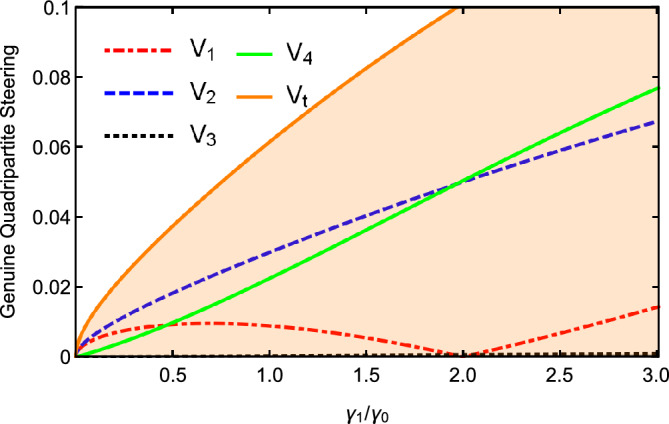
The values of $V_{i}$ and $V_{t}$ versus $\gamma _{1}/\gamma _{0}$, with $\gamma _{0}=0.02$, $\gamma _{2}=2\gamma _{1}$, $\gamma _{3}=4\gamma _{1}$ , $\gamma _{4}=5\gamma _{1}$, $\kappa _{1}=5\gamma _{1}$, $\kappa _{2}=0.5\kappa _{1}$, and $\ \kappa _{3}=0.2\kappa _{1}$ below the threshold.

The influences of the damping rate $\gamma _{1}/\gamma _{0}$ on the $V_{i}$ and $V_{t}$ are plotted as a function in Fig. 4 with $\varepsilon =0.08\varepsilon _{c}$, $\gamma _{0}=0.02$, $\gamma _{2}=2\gamma _{1}$, $\gamma _{3}=4\gamma _{1}$, $\gamma _{4}=5\gamma _{1}$, $\kappa _{1}=5\gamma _{1}$, $\kappa _{2}=0.5\kappa _{1}$, and $\ \kappa _{3}=0.2\kappa _{1}$. From Fig. 4, one can see that the values of $V_{i}$ and $V_{t}$ increase with the increase of the damping rates. When $\gamma _{1}/\gamma _{0}>2$, $V_{t}>1$, the quadripartite quantum steering can not be obtained by the cascaded nonlinear process. The damping rate of parametric optical field is smaller than that of pump light, so that all optical fields can resonate in the cavity. A smaller damping rate of parametric optical field can obtain a better multipartite quantum steering correlation.

**Figure 5:**
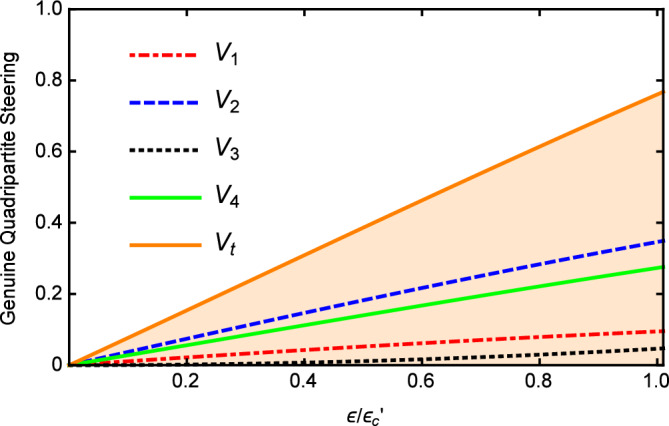
The values of $V_{i}$ and $V_{t}$ versus $\varepsilon$, with $\gamma _{0}=\gamma _{1}=0.02$, $\gamma _{2}=2\gamma _{1}$, $\gamma _{3}=4\gamma _{1}$ , $\gamma _{4}=5\gamma _{1}$, $\kappa _{1}=5\gamma _{1}$, $\kappa _{2}=0.5\kappa _{1}$, and $\ \kappa _{3}=0.2\kappa _{1}$ below the threshold.

Figure 5 shows that the values of *Vi* and $V_{t}$ versus $\varepsilon$ with $\gamma _{0}=\gamma _{1}=0.02$, $\gamma _{2}=2\gamma _{1}$, $\gamma _{3}=4\gamma _{1}$, $\gamma _{4}=5\gamma _{1}$, $\kappa _{1}=5\gamma _{1}$, $\kappa _{2}=0.5\kappa _{1}$, $\kappa _{3}=0.2\kappa _{1}$, and $\omega =5\gamma _{0}$. It can be clearly seen that the value of $V_{i}$ is below 1 and most importantly, $V_{t}$ is also below 1 in the whole range, which demonstrates the success of the quadripartite EPR steering again. With the increase of pump power, the values of *Vi* and $V_{t}$ increase linearly. When the pump is weak, a better multipartite EPR steering can be obtained. It can be seen from the above analysis, the genuine quadripartite EPR steering can be generated below the threshold by cascaded nonlinear processes in our scheme.

**Figure 6:**
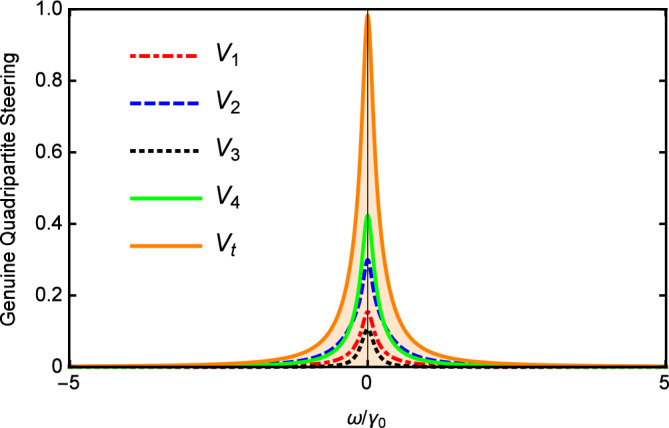
The values of $V_{i}$ and $V_{t}$ versus $\Omega$, with $\gamma _{0}=0.1$, $\gamma _{1}=\gamma _{3}=0.2 \gamma _{0}$, $\gamma _{2}=0.4\gamma _{0}$, $\gamma _{4}=0.1\gamma _{0}$, $\kappa _{1}=0.1\gamma _{0}$, $\kappa _{2}=4\kappa _{1}$, $\kappa _{3}=2 \kappa _{1}$ without the threshold.

**Figure 7:**
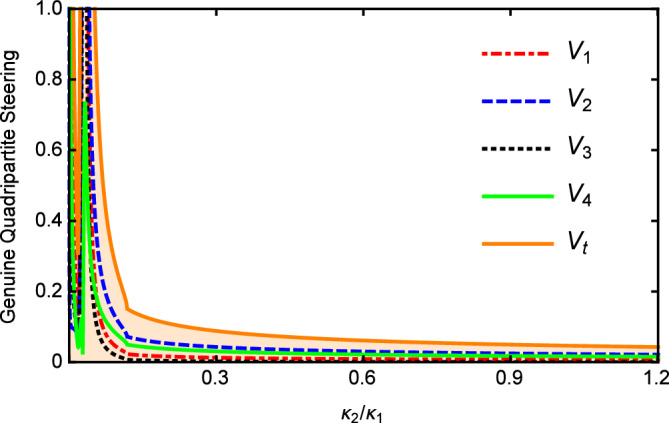
The values of $V_{i}$ and $V_{t}$ versus $\kappa _{2}/\kappa _{1}$, with $\gamma _{0}=0.1$, $\gamma _{1}=\gamma _{3}=0.2\gamma _{0}$, $\gamma _{2}=0.4\gamma _{0}$, $\gamma _{4}=0.1\gamma _{0}$, $\kappa _{1}=0.1\gamma _{0}$, $\kappa _{3}=2\kappa _{1}$ without the threshold.

### Without the threshold

In the condition of without oscillation threshold, only when $\varepsilon <\varepsilon _{c}^{^{\prime }}$ the system is stable and the the linearization method is effective. Figure 6 depicts $V_{i}$ and $V_{t}$ versus the normalized analysis frequency $\Omega =\omega /\gamma _{0}$ for $\gamma _{0}=0.1$, $\gamma _{1}=\gamma _{3}=0.2\gamma _{0}$, $\gamma _{2}=0.4\gamma _{0}$, $\gamma _{4}=0.1\gamma _{0}$, $\kappa _{1}=0.1\gamma _{0}$, $\kappa _{2}=4\kappa _{1}$, $\kappa _{3}=2\kappa _{1},$ and $\varepsilon =0.5\varepsilon _{c}^{^{\prime }}$. The curves of $V_{i}$ and $V_{t}$ are below 1 in the whole range of $\Omega$ which shows that the genuine quadripartite EPR steering can be generated in the case of without the threshold.

Figure 7 shows the effects of the nonlinear coupling parameter $\kappa _{2}/\kappa _{1}$ on $V_{i}$ and $V_{t}$ with $\gamma _{0}=0.1$, $\gamma _{1}=\gamma _{3}=0.2\gamma _{0}$, $\gamma _{2}=0.4\gamma _{0}$, $\gamma _{4}=0.1\gamma _{0}$, $\kappa _{1}=0.1\gamma _{0}$, $\kappa _{3}=2\kappa _{1}$, and $\varepsilon =0.5\varepsilon _{c}^{^{\prime }}$. One can see that when $\kappa _{2}<0.1\kappa _{1}$, the values of $V_{i}$ and $V_{t}$ are all above 1 and there is a sharp decline. In this case, the quadripartite EPR steering can not be obtained. However, when $\kappa _{2}>0.1\kappa _{1}$, it is clearly see that $V_{i}$ and $V_{t}$ are all below 1, and the quadripartite EPR steering are present.

**Figure 8:**
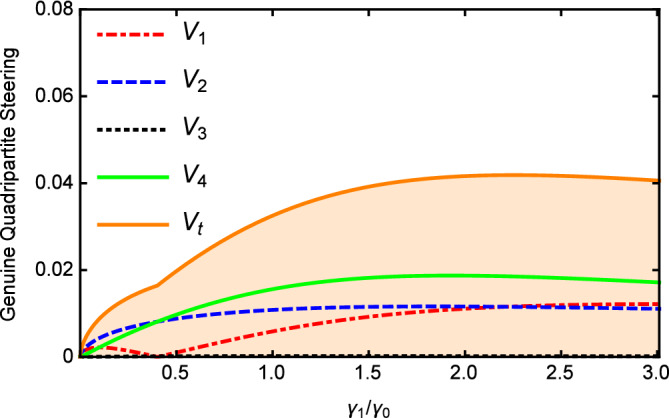
The values of $V_{i}$ and $V_{t}$ versus $\gamma _{1}/\gamma _{0}$, with $\gamma _{0}=0.1$, $\gamma _{3}=0.2\gamma _{0}$, $\gamma _{2}=0.4\gamma _{0}$, $\gamma _{4}=0.1\gamma _{0}$, $\kappa _{1}=0.1 \gamma _{0}$, $\kappa _{2}=4\kappa _{1}$, $\kappa _{3}=2\kappa _{1}$ without the threshold.

Figure 8 describes $V_{i}$ and $V_{t}$ versus the damping rates $\gamma _{1}/\gamma _{0}$ with $\gamma _{0}=0.1$, $\gamma _{2}=0.4\gamma _{0}$, $\gamma _{3}=0.2\gamma _{0}$, $\gamma _{4}=0.1\gamma _{0}$, $\kappa _{1}=0.1\gamma _{0}$, $\kappa _{2}=4\kappa _{1}$, $\kappa _{3}=2\kappa _{1}$, and $\varepsilon =0.5\varepsilon _{c}^{^{\prime }}$. As shown in Fig. 8, the values of $V_{i}$ and $V_{t}$ are all below 1 in whole range, which also demonstrates the success of the quadripartite EPR steering.

**Figure 9:**
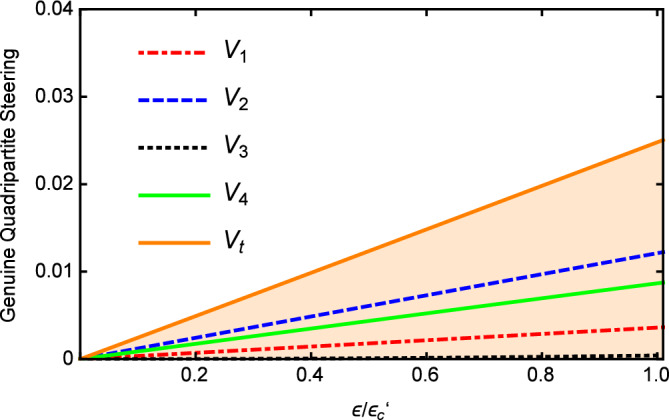
The values of $V_{i}$ and $V_{t}$ versus $\varepsilon$, with $\gamma _{0}=0.1$, $\gamma _{1}=\gamma _{3}=0.2\gamma _{0}$, $\gamma _{2}=0.4\gamma _{0}$, $\gamma _{4}=0.1\gamma _{0}$, $\kappa _{1}=0.1 \gamma _{0}$, $\kappa _{2}=4\kappa _{1}$, $\kappa _{3}=2\kappa _{1}$ without the threshold.

Finally, the effects of changing pump value $\varepsilon$ on the $V_{i}$ and $V_{t}$ with $\gamma _{0}=0.1$, $\gamma _{1}=\gamma _{3}=0.2\gamma _{0}$, $\gamma _{2}=0.4\gamma _{0}$, $\gamma _{4}=0.1\gamma _{0}$, $\kappa _{1}=0.1\gamma _{0}$, $\kappa _{2}=4\kappa _{1}$, $\kappa _{3}=2\kappa _{1}$ is plotted in Fig. 9. It is shown that the values of the $V_{i}$ and $V_{t}$ are all below 1 and a better multipartite EPR steering can be obtained for weaker pump which is same to the case in Fig. 5. This may be because its quantum properties become apparent when the pump power is weak. Generally speaking, the genuine quadripartite EPR steering can be confirmed when the system without oscillation threshold.

## Discussion

SPDC cascaded with double sum-frequency generations in an optical cavity is investigated below the oscillation threshold and without oscillation threshold, respectively. According to the criterion for the genuine multipartite EPR steering^4–6^, it is confirmed that the genuine quadripartite EPR steering can be generated in the regimes below the oscillation threshold and without oscillation threshold. The effects of nonlinear parameters on quadripartite EPR steering are also discussed. The present scheme of the generation of quadripartite EPR steering is different from the previous schemes of the generation of triple-photon states quantum entanglement and steering such as in Refs.^29,30^. Only tripartite quantum steering or entanglement can be obtained by one nonlinear process of three-photon SPDC in their scheme. In our present scheme, quadripartite quantum steering can be obtained by three cascaded nonlinear processes of SPDC cascaded two sum-frequency processes. It is also different from our previous scheme of quadripartite quantum steering in Ref.^22^. Only one sum-frequency generation process was considered and there was an injected signal for the optical cavity in the previous study. Moreover, the threshold characteristics of cascaded nonlinear process was not discussed in the previous scheme. In present scheme, based on the threshold characteristics of the system, we find that the system is unstable when considering the quantum nature of pump which is very different from the case in Ref.^22^. Therefore, we will investigate the multipartite quantum steering among parametric and sum-frequency optical fields excluding pump. In this way, one can discover under what conditions stable quadripartite quantum steering can be obtained.

The damping rates $\gamma _i$ is related to the reflection transmission coefficient of the optical cavity for $t_i=\sqrt{2\gamma _i}$. For example, $\gamma _i$=0.02, the transmittance of the coupling mirror to the optical field is $t^2_i=2\gamma _i=0.04\%$ and the reflectivity is $r^2_i=96\%$. The nonlinear coupling parameter $\kappa$ is related to pump power, nonlinear polarizability, and the structure parameters of optical superlattice. In our present scheme, we can change the nonlinear coupling parameter $\kappa$ by designing the parameters of optical superlattice. We think that the theoretical research results in this study can provide reference data for experiments.

SPDC cascaded one sum-frequency process has been realized in quasi-periodic optical superlattice in the experiments^31,32^. SPDC cascaded two double-frequency processes of signal and idler has also been achieved in experiment by QPM technique^33^. In addition, based on the experiment^34^, we think it should be possible to realize the simultaneous resonance of all the beams in the cavity. Therefore, SPDC cascaded two sum-frequency processes in our present scheme is experimentally feasible. The present scheme provides a concrete in-depth understanding of EPR steering in experiment and has potential applications in quantum information.

## Data Availability

The data that support the findings of this study are available from the corresponding author upon request.
